# Screening the Reference Genes for Quantitative Gene Expression by RT-qPCR During SE Initial Dedifferentiation in Four *Gossypium hirsutum* Cultivars that Have Different SE Capability

**DOI:** 10.3390/genes10070497

**Published:** 2019-06-28

**Authors:** Aiping Cao, Dongnan Shao, Baiming Cui, Xuecheng Tong, Yinying Zheng, Jie Sun, Hongbin Li

**Affiliations:** 1 Key Laboratory of Xinjiang Phytomedicine Resource and Utilization of Ministry of Education, College of Life Sciences, Shihezi University, Shihezi 832003, China; 2 Key Laboratory of Oasis Eco-Agriculture, Shihezi University, Shihezi 832003, China

**Keywords:** reference gene, quantitative gene expression, RT-qPCR, *Gossypium hirsutum*, somatic embryogenesis

## Abstract

RNA sequencing (RNA-Seq)-based gene expression analysis is applicable to a wide range of biological purposes in various species. Reverse transcription quantitative PCR (RT-qPCR) is also used to assess target gene expression utilizing stably expressed reference genes as internal control under a given set of conditions. However, investigations of the reference genes for RT-qPCR normalization in the process of somatic embryogenesis (SE) initial dedifferentiation in *Gossypium hirsutum* are rarely reported. In this study, on the basis of our previous transcriptome data of three different induction stages during SE initial dedifferentiation process in four *G. hirsutum* cultivars that have different SE capability, 15 candidate genes were selected during SE initial dedifferentiation process, and their expression stability was evaluated by geNorm, NormFinder, and BestKeeper. The results indicated that the two genes of *endonuclease 4* (*ENDO4*) and *18S ribosomal RNA* (*18S rRNA*) showed stable expression in the four different *G. hirsutum* cultivars, endowing them to be appropriate reference genes during three induction stages in the four cotton cultivars. In addition, the stability and reliability of the two reference genes of *ENDO4* and *18S rRNA* were further verified by comparing the expressions of *auxin-responsive protein 22* (*AUX22*) and *ethylene-responsive transcription factor 17* (*ERF17*) between RT-qPCR results and the RNA-seq data, which showed strong positive correlation coefficient (R^2^ = 0.8396–0.9984), validating again the steady expression of *ENDO4* and *18S rRNA* as the reliable reference genes. Our results provide effective reference genes for RT-qPCR normalization during SE process in different *G. hirsutum* cultivars.

## 1. Introduction

Gene expression level analysis is crucial in many fields of biological research [1]. Currently, RNA sequencing (RNA-Seq) has become the prevalent method used to analyze the transcriptional gene expression levels in various species [2,3]. Reverse transcription quantitative PCR (RT-qPCR) is the widely applied method to quantify gene expression and to validate transcriptomic data [4,5] for its prominent advantages of high sensitivity, reproducibility, and specificity [6]. However, inaccurate quantification and low-quality RNA may lead to a decrease in RT-qPCR specificity [7]. Thus, it is necessary and crucial to screen stably expressed reference genes to normalize target gene expression level under a given set of conditions. Reliable reference genes are usually selected from the stably expressed genes that are associated with basic cellular function and cell processes, including cell structure formation, cytoskeletal protein formation, and ribosomal subunit synthesis [1,8,9].

The expression levels of some reference genes fluctuate under diverse conditions in different species. The *elongation factor 1-alpha* (*EF1α*) and *beta-tubulin 3* (*TUB3*) would be suitable for normalizing gene expression data under salt and drought stresses, respectively, while *ubiquitin carrier protein 10* (*UBC10*) appeared to be a better option under the coaction of the two stresses in *Halostachys caspica* [9]. In *Caragana intermedia*, *hypothetical protein 2* (*UNK2*), *protein phosphatase 2A* (*PP2A*), and *sand family protein gene* (*SAND*) were the most suitable and stable reference genes across all tested conditions, while *UNK2*, *SAND,* and *EF1α* were suitable for salt-treated leaves, and *UNK2* and *SAND* were proper for salt-treated roots, respectively. Additionally, the reference genes, *tonoplast intrinsic protein 41* (*TIP41*) and *PP2A* for polyethylene glycol (PEG)- treated leaves; *hypothetical protein 1* (*UNK1*)*, UNK2,* and *PP2A* for PEG-treated roots; *SAND* and *EF1α* for cold-treated leaves; *SAND*, *TIP41,* and *PP2A* for heat-treated leaves, were discovered as suitable candidates in different treated cotton tissues [3]. In *Gracilaria lemaneiformis* subjected to various temperature stimulations, *glyceraldehyde-3-phosphate dehydrogenase* (*GAPDH*), *isoleucine-tRNA synthetase* (*ITS2*)*, compensatory Response* (*CR*), and *18S rRNA* were the optimal reference genes for various treatments applied at 8°C, while *eukaryotic translation initiation factor* (*eIF*) and *actin* (*ACT*) were appropriate for treated materials at 32°C, and *GAPDH*, *EF1α,* and *ACT* were ideal for different temperature treatments [10]. In *Hedera helix*, *ribosomal 40S* (*40S*) was the suitable reference gene after abscisic acid (ABA) treatment, while under various cold stress conditions in different tissues, *squalene epoxidase* (*SE*) and *beta-amyrin synthase* (*β-AS*) were perfected as reference genes [11].

Cotton fibers are major raw materials for the textile industry, and seeds are an important source of oil. Somatic embryogenesis (SE) has been considered practically as a tool for clonal propagation of cotton and is often used for the genetic modification of cotton in concert with *Agrobacterium*-mediated transformation, providing the effective basis for further investigating the gene function and understanding the regulatory mechanism in cotton plants at the molecular, cellular, and tissue levels [12]. Nonetheless, only a few cotton varieties have been successfully genetically modified in vitro using SE [13]. The majorities of the cotton cultivars are difficult to regenerate via SE [14], and many factors may affect SE success, including culture conditions and tissue background. Therefore, to date, most studies have focused on analyzing the molecular mechanism and identifying the genes critical for SE [15,16]. Although some miRNAs have been reported as reference genes in different cotton tissues [17,18], no systematic analysis was investigated for reference genes applied to be as a control in SE development through RT-qPCR method. 

On the basis of our previous RNA-Seq data of *Gossypium hirsutum*, we selected 15 candidate reference genes, of which *ENDO4* and *18S rRNA* genes appeared to be stably expressed in the four *G. hirsutum* cultivars that have different SE capability after evaluation by the software geNorm [19], NormFinder [20], and BestKeeper [21] and verification of the expression consistency between the RT-qPCR results and the RNA-Seq data. In addition, the genes of *ethylene-responsive transcription factor 17 (ERF17)* and *auxin-responsive protein 22 (AUX22)* with altered expressions were further selected to validate the expression stability of *ENDO4* and *18S rRNA*, showing again the highly steady expression of the two genes. All these results suggest that *ENDO4* and *18S rRNA* can be used as appropriate reference genes for RT-qPCR normalization during SE initial dedifferentiation in different *G. hirsutum* cultivars. 

## 2. Materials and Methods 

### 2.1. Plant Materials

Four *G. hirsutum* cultivars, YZ1, R15, X33, and X42, were chosen as the cotton materials. Of which, YZ1 and R15 have a relatively high SE differentiation rate and, therefore, were commonly used as the main transgenic materials [22]. Although being the major commercial cultivars in Xinjiang, China, X33 and X42 have a low rate of differentiation during SE [23]. 

### 2.2. Total RNA Extraction and cDNA Synthesis

Total RNA extracted from different cotton tissues were subjected to check the purity using a NanoPhotometer spectrophotometer (IMPLEN, Calabasas, CA, USA), with the concentration and integrity measured by a Qubit® 2.0 Fluorometer (Life Technologies, Carlsbad, CA, USA) and the Bioanalyzer 2100 system (Agilent Technologies, Carlsbad, CA, USA). The cDNA was synthesized from each RNA sample using the PurelinkTM RNA Mini Kit (Life Technologies, Carlsbad, CA, USA) and the PrimerScript RT reagent Kit (Takara, Shiga, Japan) following the manufacturer’s protocols. The detailed steps were established according to our previously described method [23]. 

### 2.3. Selection of Reference Genes and Design of Primers 

We performed high throughput RNA-Seq on four cultivars of *G. hirsutum* at three different induction stages (0h, 3h, and 3d). The four cultivars have different SE differentiation rate. Hypocotyl of 6-d sterile seedlings was cut into 1 cm segments with successive induction at 0 h, 3 h, and 3 d on callus-induction medium (MS medium plus B5 vitamins, supplemented with 0.05 mg/L IAA, 0.05 mg/L kinetin, 0.05 mg/L 2,4-D, pH 5.8) [23]. To evaluate the gene expression stability, q-value≥0.05, FPKM≥5, and |log2FoldChange|<1 were used as the criteria for screening reference genes at all sampling points [24], and 15 reference genes were selected. Gene-specific primers were designed based on the sequences of the 15 genes using the online software NCBI/Primer-BLAST (https://www.ncbi.nlm.nih.gov/tools/primer-blast/) according to the following parameters: PCR product size of 100–150 bp, primer melting temperature (Tm) of 57°C–60°C, and primer pairs separated by at least one intron on the corresponding genomic DNA [25]. All primer pairs were synthesized by BGI TECH (BGI TECH, Shenzhen, China), and the PCR products were verified with electrophoresis on 1% agarose gels. 

### 2.4. RT-qPCR Analysis

RT-qPCR was executed in 96-well plates on a LightCycler® 480 Real-Time PCR System (Roche Diagnostics, Mannheim, Germany) with an SYBR Green-based PCR assay. Reactions with a total volume of 10 μL, including 1 μL of a template (first-strand cDNA), 0.4 μL each of 10 μM forward and reverse gene-specific primers, 5 μL of 2×SYBR Premix Ex Taq II (TLi RanseH Plus) (Takara, Dalian, China), and 3.2 μL of ddH_2_O. The RT-qPCR conditions were as follows: initial denaturation at 95°C for 30 s, followed by 45 cycles of 95°C for 10 s, 60°C for 10 s, and 72°C for 10 s. The RT-qPCR analysis was tested in three biological replicates. Additionally, three technical replicates were used for each RT-qPCR analysis, and the R^2^ and amplification efficiency (E) were counted by the standard curves with the diluted series on the basis of the diluted cDNA series (version 1.5, Roche Diagnostics, Mannheim, Germany). The PCR efficiency was detected by the equation (E = (10^[−1/slope]^ − 1) × 100%) [19].

### 2.5. Data Analysis

The raw Cq (quantification cycle) values are listed in Supplementary Table S1. Three common software programs, geNorm, NormFinder, and BestKeeper, were applied to calculate the expression stability of candidate reference genes. For geNorm and NormFinder, Cq values were converted into relative quantities according to the formula: 2^−ΔCt ^(ΔCt = the corresponding Cq value − minimum Cq) [26]. The BestKeeper calculations were directly based on raw Cq values of each gene. 

### 2.6. Validation of Reference Genes

To test the stability and reliability of the reference genes, the results of RT-qPCR were compared with the RNA-Seq data. The expression profiles of *ERF17* and *AUX22* were analyzed and compared with the values of Fragments Per Kilobase of transcript sequence per millions of base pairs sequenced (FPKM) obtained from the RNA-Seq data. The RT-qPCR analysis was conducted in the three biological replicates. Data were analyzed using the Origin 8 software and R package.

## 3. Results

### 3.1. Isolation of Candidate Reference Genes in Different *G. hirsutum* Cultivars 

**Figure 1:**
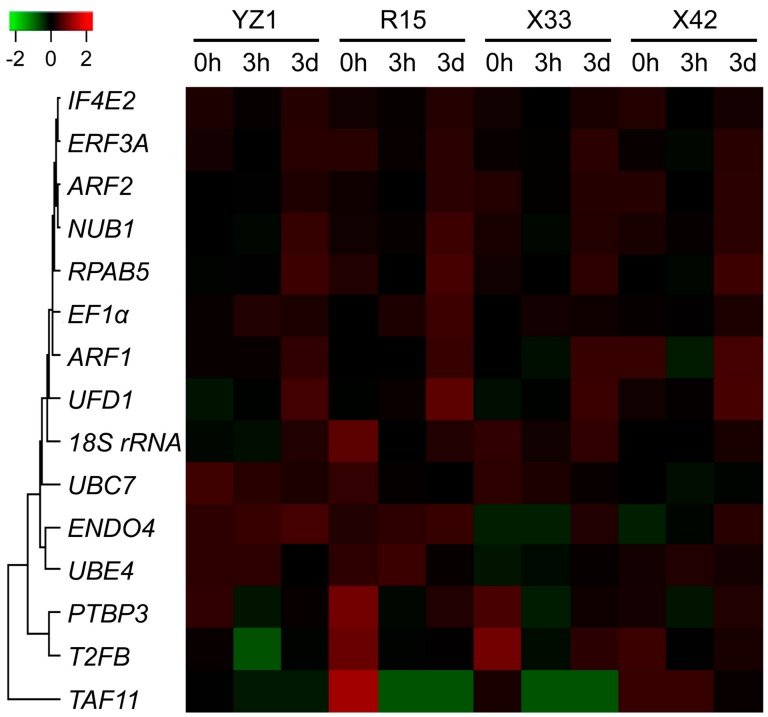
FPKM-based heatmap of the 15 candidate reference genes during three induction stages in four cotton cultivars. Fifteen candidate reference genes were selected from the *Gossypium hirsutum* transcriptome datasets with the screening conditions of q-value ≥ 0.05, FPKM ≥ 5, and |log2FoldChange| < 1. Higher or lower FPKM of candidate reference genes were colored by red or green in four cotton cultivars, respectively. The color bar was shown at the upper apex. The visualized heatmap was generated using the R package based on the FPKM from the *G. hirsutum* transcriptome datasets. The sequences of the 15 candidate reference genes, including *18S rRNA*, *ENDO4*, *EF1α*, *ADP-ribosylation factor 1* (*ARF1*), *ADP-ribosylation factor 2* (*ARF2*), *eukaryotic peptide chain release factor 3A* (*ERF3A*), *eukaryotic translation initiation factor isoform 4E-2* (*IF4E2*), *NEDD8 ultimate buster 1* (*NUB1*), *polypyrimidine tract-binding protein homolog 3* (*PTBP3*), *RNA polymerases I, II, and III subunit* (*RPAB5*), *transcription initiation factor (IIF), beta subunit* (*T2FB*), *TBP-associated factor 11* (*TAF11*), *U-box domain-containing protein* (*UBE4*), *ubiquitin carrier protein 7* (*UBC7*), and *ubiquitin fusion degradation 1* (*UFD1*), were obtained from NCBI GenBank nucleotide sequence database with the detailed information of the gene names and the corresponding accession numbers provided in Table 1.

According to our previous RNA-Seq-based transcriptome data of three different induction stages during SE initial dedifferentiation process in four *G. hirsutum* cultivars that have different SE capability, the screen rules of q-value ≥ 0.05, FPKM ≥ 5, |log2FoldChange| < 1, and relatively lower coefficient of variance (CV) of FPKM were set as the higher criteria at all sampling points to select the candidate reference genes. A total of 15 candidate reference genes were obtained, with the details of all reference genes listed in Supplementary Table S2 and the heatmap of FPKM during three induction stages in four cotton cultivars shown in Figure 1. 

### 3.2. Verification of Primer Specificity and PCR Amplification Efficiency

The 15 candidate reference genes were amplified by RT-qPCR using cDNA as the template to verify the specificity of the primers. One distinctive peak was observed in each melting curve, indicating that all the specific primers were appropriate for further RT-qPCR detection (Supplementary Figure S1). Gel electrophoresis results indicated that all specific primers could obtain a corresponding PCR product as designed in Table 1 successfully. The RT-qPCR amplification efficiency and R^2^ of the candidate reference genes were calculated based on the slopes of the standard curves in the four cotton cultivars, which showed that the RT-qPCR amplification efficiency ranged from 96.13% (*TAF11*) to 120.30% (*UBE4*), and the R^2^ values ranged from 0.9677 to 1.0977 (Table 1), suggesting that the primers had high specificity and amplification efficiency and were appropriate for RT-qPCR. 

### 3.3. Expression Profile Analysis of the Candidate Reference Genes at Different Induction Stages in Different Cotton Cultivars

**Figure 2:**
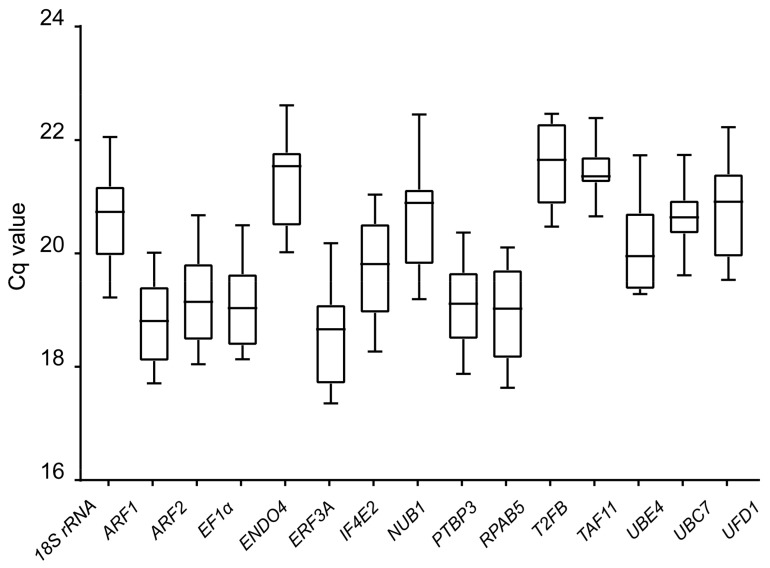
Cq (quantification cycle) values of the 15 candidate reference genes across all samples. The final Cq value of each cotton sample was the mean of three biological and technical replicates. Box graph indicates the interquartile range. A line across the box represents the median. Whisker caps represent the maximum and minimum values.

RT-qPCR was used to measure the gene expression levels of the 15 candidate reference genes at three different induction stages in the four different cotton cultivars. Gene expression level was calculated based on the quantification cycle (Cq) that referred to the amplification-related fluorescence to reach a specific threshold level of test, with a smaller Cq representing a higher gene expression. Fifteen reference genes displayed a relatively wide range of mean Cq values from 17.36 to 22.61 (Figure 2, Supplementary Table S1). The most highly expressed gene was *ERF3A* in the X42 cultivar at the induction stage of 3 d, while *END04* showed the lowest level in R15 at the induction stage of 0 h. Additionally, *18S rRNA* showed the least gene expression variation, while *UBE4* showed the greatest variation among the different cotton cultivars.

### 3.4. Expression Stability Analysis of Reference Genes 

**Figure 3:**
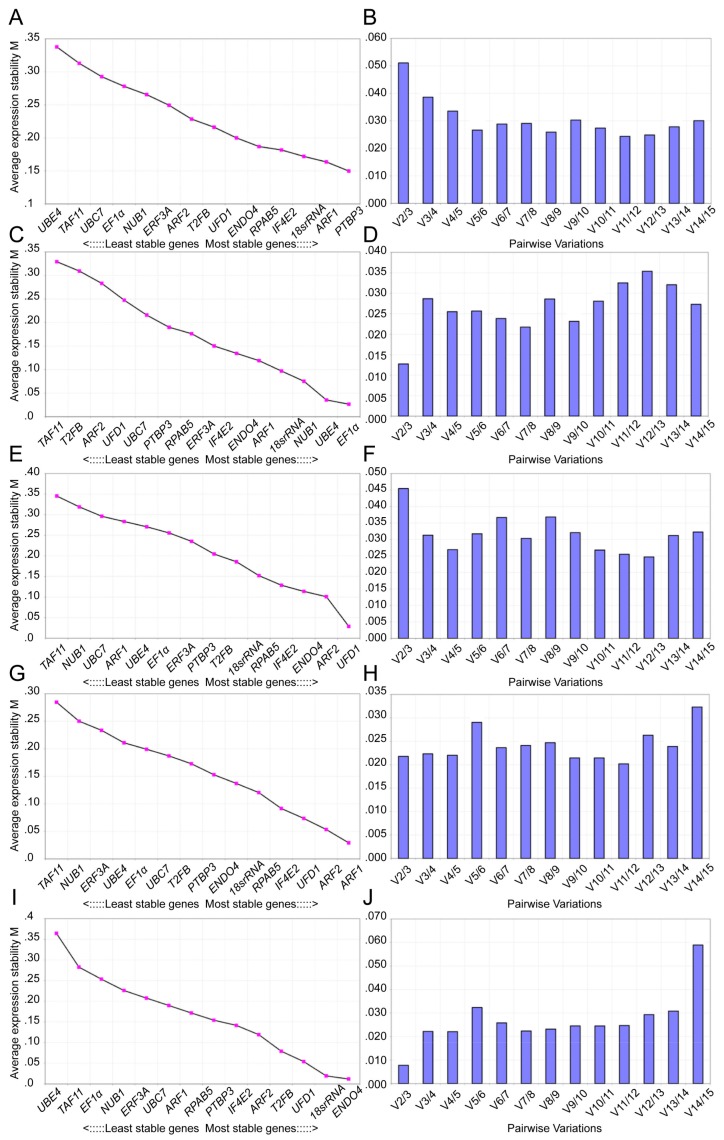
geNorm analysis of the values of average expression stability (M) and pairwise variation (Vn/Vn+1) for the candidate reference genes. M and pairwise variation (Vn/Vn+1) were calculated on the basis of 2−ΔCt (ΔCt = the corresponding Cq value – minimum Cq) by geNorm. Lower M values indicate more stable gene expression. Ranking of the gene expression stability was determined in presented samples. According to the algorithm and software instructions, the cut-off V value of 0.15 was used to determine the optimal number of reference genes for RT-qPCR normalization. (**A–B**) Values of M and Vn/Vn+1 for the reference genes in the four cotton cultivars. (**C–D**) Values of M and Vn/Vn+1 for the reference genes in YZ1. (**E–F**) Values of M and Vn/Vn+1 for the reference genes in R15. (**G–H**) Values of M and Vn/Vn+1 for the reference genes in X33. (**I–J**) Values of M and Vn/Vn+1 for the reference genes in X42.

To identify the stably expressed gene for cotton RT-qPCR normalization, the gene expression stability was assessed using three publicly available statistical tools, geNorm, NormFinder, and BestKeeper. geNorm can be used to check the most suitable number of reference genes by estimating M value that corresponds to the stability, with M = 1.5 or <1.5 indicating the standard or increased degree of stability of the candidate reference genes [19]. As shown in Table 2 and Figure 3, the results of the geNorm analysis showed that *18S rRNA*, *END04,* and *ARF1* were the most suitable reference genes in the four different cotton cultivars at three induction stages, while *UBC7* and *UBE4* were the least suitable reference genes. For the cotton cultivar YZ1, *ARF1* had the lowest M values, and *TAF11* had the highest M values under the same conditions. *END04*, *IF4E2,* and *18S rRNA* in R15, *END04, ARF2,* and *ARF1* in X33, and *ARF2*, *T2FB,* and *UFD1* in X42 were detected as the more stably expressed genes, respectively. The variation V/(V_n/n+1_) between gene pairs can also establish the optimal number of reference genes for normalization. Generally, V_n/n+1_ is the cut-off value. If V_n/n+1_ = 0.15, the optimal number of reference genes for accurate normalization should be optimal n+1; if V_n/n+1_ < 0.15, the optimal number of reference genes should be n [27]. As shown in Figure 3, the V_2/3_ value was below 1.5; in conclusion, according to the geNorm analysis, the most stable reference genes were *18S rRNA* and *END04* in four cotton cultivars.

The NormFinder software is a Visual Basic application tool that establishes the expression stability of reference genes based on the stability value (Sv). The NormFinder analysis differs slightly from geNorm analysis, which takes into account intra- and inter-group variation when calculating the normalization factor (NF) [20]. Genes that have lower levels than an average of expression stability are more stable, and thus may be suitable as reference genes. As shown in Table 3, the results of NormFinder analysis were highly consistent with those of the geNorm analysis. The most stable genes were *18S rRNA*, *END04,* and *ARF1* in four cotton cultivars. *PRAB5*, *ARF1,* and *18S rRNA* wer*e* the most stable genes in YZ1. *END04*, *IF4E2,* and *18S rRNA* had high expression stabilities in R15. For the cotton cultivar X33, *END04*, *ARF2,* and *18S rRNA* appeared to have the most stable expressions. *ARF2*, *T2FB,* and *UFD1* were highly stably expressed in X42. 

The BestKeeper software can be used to analyze the stability and expression of reference genes based on the coefficient of variance (CV), standard deviation (SD), and correlation coefficient (R) [21]. Reference genes are considered to be stable if R is high, but SD and CV are low. Additionally, if SD > 1, the gene is considered to be unacceptable [9]. As shown in Table 4, *END04*, *18S rRNA,* and *ARF1* were relatively stable expressed genes under all conditions. *TAF11* was an unstable gene with a low CV± SD value and lower R value. For R15 cotton cultivar, *18S rRNA, UBC7,* and *ARF1* were identified as relatively suitable reference genes. *END04*, *18S rRNA,* and *PTBP3* in X33 and *T2FB*, *ARF2,* and *EDD04* in X44 were the more stably expressed genes and could be considered as acceptable reference genes with high R values and low CV ± SD values. These results of comprehensive consideration of the geNorm, NormFinder, and BestKeeper analyses indicate that *18S rRNA* and *ENDO4* could be appropriate reference genes.

### 3.5. Expression Stability Validation of the Reference Genes of 18S rRNA and ENDO4 

**Figure 4:**
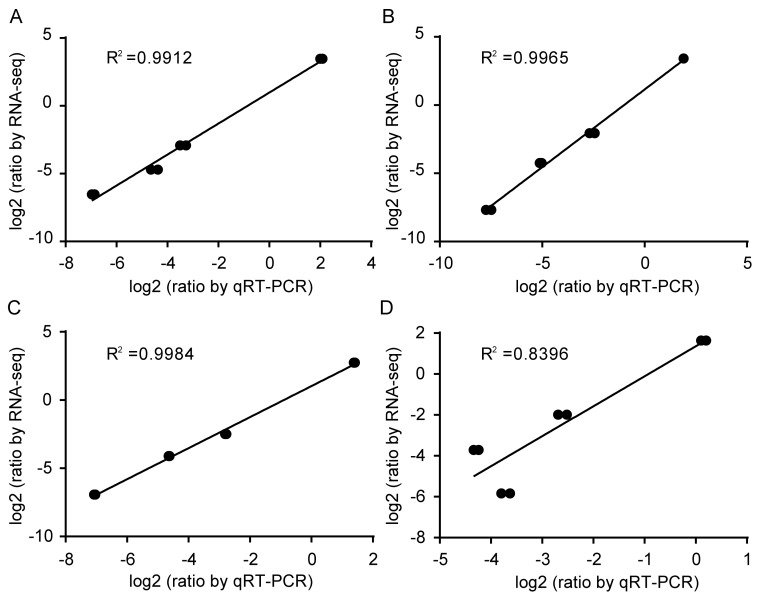
Correlation analysis between RT-qPCR results and the RNA-Seq data. *X*-axis represented log2(ratio by RT-qPCR), and equation 2^−ΔCt^ was applied to calculate the RT-qPCR relative expression level using selected target genes as the reference gene. *Y*-axis represented log2(ratio by RNA-Seq) (*p*-value ≤ 0.01). Correlation analysis using *ENDO4* and *18S rRNA* was used as the internal controls for normalization of *AUX22* and *ERF17* in YZ1 (**A**), R15 (**B**), X33 (**C**), and X42 (**D**).

**Figure 5:**
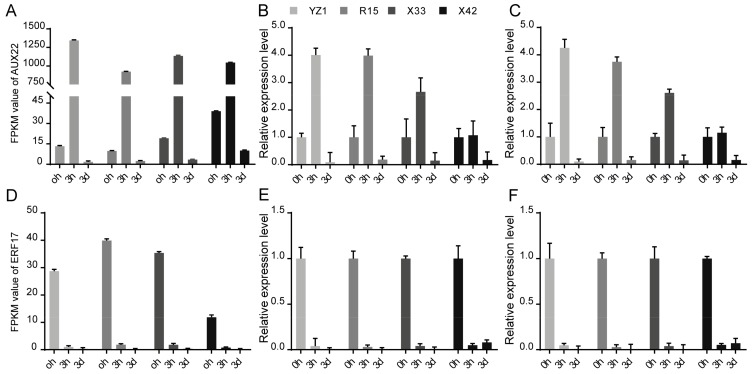
Validation of the reference genes of *18S rRNA* and *ENDO4* as internal controls for normalization of target gene expressions of *AUX22* and *ERF17*. The results were shown as mean fold changes in relative expression when compared to 0 h. (**A**) The FPKM values of *AUX22* in RNA-Seq data. (**B**) The expression profiles of *AUX22* normalized using *18S rRNA* as an internal control. (**C**) The expression profiles of *AUX22* normalized using *ENDO4* as an internal control. (**D**) The FPKM values of *ERF17* in RNA-Seq data. (**E**) The expression profiles of *ERF17* normalized using *18S rRNA* as an internal control. (**F**) The expression profiles of *ERF17* normalized using *ENDO4* as internal control.

Through the identity analysis between the RT-qPCR results and the RNA-Seq data, two target genes, *AUX22* and *ERF17,* were selected to further test the stability and reliability of the validated reference genes of *18S rRNA* and *ENDO4*. The relative expression levels of *AUX22* and *ERF17* were calculated based on the validated reference genes and then were compared with the relative expression levels and the FPKM values of these reference genes in the RNA-Seq data. The *18S rRNA* and *ENDO4* were used as reference genes for the normalization of the target gene expression in the four cotton cultivars (Figure 4). Strong positive correlation coefficients (R^2^ = 0.8396–0.9984) between the RT-qPCR results and the RNA-Seq data, and similar patterns between relative expression profiles and the FPKM values, were also observed (Figure 5). The results indicated that the *18S rRNA* and *ENDO4* were suitable as the reference genes to obtain the reliable RT-qPCR results in the four cotton cultivars.

## 4. Discussion

Gene expression analysis is an effective basic method to predict plant gene function [28]. RT-qPCR is also widely used for gene expression detection using proper reference gene as an internal control [6]. Whereas, the expression of the reference gene is unavoidably influenced by different tissues and treatments, thus leading to unreliable results [29]. Therefore, it is essential to select valid and reliable reference genes as internal controls for normalization to ensure the reliability and accuracy of RT-qPCR reactions, without consideration of different experimental conditions [8]. In this study, on the basis of our high throughout RNA-Seq data of four *G. hirsutum* cultivars at three induction stages, we analyzed 15 candidates as reference genes according to the conditions of |log2FoldChange|<1 and a small coefficient of variation (CV) of FPKM values (Figure 1), which are widely considered parameters for choosing candidate reference genes [30]. 

Three public statistical algorithms, geNorm, NormFinder, and BestKeeper, can be used to determine the stability of reference gene expression [19,20,21]. Some studies of the two programs of geNorm and NormFinder were mostly used to identify candidate reference genes, showing the results that differed slightly in *Oxytropis ochrocephala* [30]. Several reports also showed that the NormFinder results were consistent with geNorm results in a variety of organisms, including the *Sapium sebiferum* [31], *Chrysanthemum morifolium* and *Chrysanthemum lavandulifolium* [32], *Oryza sativa* [33], and *Rhododendron molle* [34]. In this study, the ranking of NormFinder was relatively consistent with the results of geNorm analysis (Table 2 and Table 3). However, the results differed from those obtained using BestKeeper, indicating that *UBC7* was the most reliable reference gene in R15 analyzed by BestKeeper, but was the least reliable one analyzed by geNorm and NormFinder (Table 4). The divergent ranking may be caused due to the different algorithms [35]. By BestKeeper analysis, most studies utilized CV and SD of the Cq values, as well as the R values, to evaluate the stability and expression of reference genes [2,34,35]. *TAF11* showed the lowest CV± SD values across all cultivars with the lowest R values in YZ1 and X42 (Table 4) and was identified as the least reliable gene by geNorm and NormFinder (Figure 3 and Table 3). Through integrated analysis of the three programs, better accuracy for each reference gene can be obtained, and our results showed that *18S rRNA* and *ENDO4* were appropriate and reliable candidates as reference genes for RT-qPCR normalization in the four cotton cultivars under the condition of three different induction stages. *ENDO4* encodes a putative endonuclease without enzyme activity, and *18S rRNA* is part of the ribosomal RNA that constitutes the basic components of the eukaryotic cells. Ribosomal genes are often recognized as suitable reference genes [9] but are observed as the least stable expressions in peaches [36]. Generally, *EF1α* and *UBCs* were often used as reference genes in plants, such as *Platycladus orientalis* and *Caragana intermedia* [3,37], while *EF1α*, *UBC7,* and *UBE4* showed unstable expressions in the different cotton cultivars (Table 2 and Table 3).

The selected reference genes based on RNA-Seq were often further verified by RT-qPCR [38]. The RT-qPCR results were compared with that of RNA-Seq data to further validate the stability of *ENDO4* and *18S rRNA* expression, with the appearance of *ENDO4* and *18S rRNA* as reference genes in the four cotton cultivars showing strong positive R^2^ between the RT-qPCR results and the RNA-Seq data (Figure 4). When *18S rRNA* and *ENDO4* were used as the reference genes in the four cotton cultivars, the expression patterns of *ERF17* and *AUX22* indicated similar correlation between the RT-qPCR results and the RNA-Seq data (Figure 5), suggesting that *ENDO4* and *18S rRNA* are reliable reference genes for studies of gene expression normalization in these four cultivars of *G. hirsutum* under three different induction stages.

## 5. Conclusion

We selected 15 candidate reference genes for RT-qPCR normalization in different cotton cultivars at three different induction stages according to the transcriptome datasets of *G. hirsutum*. After assessment of the 15 candidates by three statistical software geNorm, NormFinder, and BestKeeper, two genes of *ENDO4* and *18S rRNA* were identified as appropriate reference genes during three induction stages in the four cotton cultivars. The results of further validation of *18S rRNA* and *ENDO4* genes by comparing the RT-qPCR results and the RNA-Seq data showed a strong positive correlation, indicating that *ENDO4* and *18S rRNA* are reliable reference genes for studies of gene expression normalization. Our results identify necessary and appropriate reference genes as internal controls for RT-qPCR normalization and provide an effective basis for accurate quantification of target gene expression in different *G. hirsutum* cultivars. 

## Figures and Tables

**Table 1:** Details of the 15 candidate reference genes, primer sequences, and amplification characteristics for RT-qPCR in *G. hirsutum*.

Gene Name	Accession Number	Primer Sequence (5′-3′)	Product Size (bp)	E^a^ (%)	R^2^
*18S rRNA*	XM_016849259	TTACGCAATGCGCTCTGGA	117	104.70	0.9968
		ACCGCAGAGCTGACAGATG			
*ARF1*	XM_016856733	CTGTGAGCAGAAAGTGGAAAGC	111	104.61	0.9996
		CAGCTGCATCAAGACCCACC			
*ARF2*	XM_016840408	CCACTTCTGGTGAAGGTCTGT	118	100.33	0.9980
		AACTCTAAAAGGGGCCAGCA			
*EF1α*	XM_016892582	CAGCTTCAGATCGCTTCTATTTCT	124	100.07	0.9998
		TGGCCAGTGGTGGTTGACTT			
*ENDO4*	XM_016854965.	TTGACAGAGGCGCTGATGTT	116	100.44	0.9905
		CTGCGGTACCAACTGACTGT			
*ERF3A*	XM_016815421	GCCCTATTTGCCACAAAACCC	140	101.56	0.9886
		TTCAGGAATGAGCGTGGCAT			
*IF4E2*	XM_016823302	CAAGACTGCAACGAATGAGGC	144	100.36	0.9894
		GCTCAAACATTGTATCGACCTTTCA			
*NUB1*	XM_016834127	TTGCACTACATATGAGGTTGGAGTT	132	101.60	0.9677
		AGGCTTCATCAGGCACTTGTA			
*PTBP3*	XM_016838300.	GTCCTTGCAAATGGCGGAAG	140	102.08	0.9961
		CCTGATTCTTTGCACGGAGC			
*RPAB5*	XM_016880466	CTTCACCTGCGGAAAGGTCA	113	99.85	1.0643
		AGCAGTACCGAACCAATCCC			
*T2FB*	XM_016852332	GGATCGCGGGGAATTGGAA	149	99.09	1.0030
		TGCCTCTCTTATTGTACACGCA			
*TAF11*	XM_016821843	TCGTCTGCATTAGAGAGTCGC	138	96.13	0.9999
		GGCTGTTCAGCTCATCCTCA			
*UBC7*	XM_016864885	GCTGGACGCCAGTACATACA	144	99.21	1.0997
		GGCTGACCTTCCTCCTGAAT			
*UBE4*	XM_016812715	TGGGCCCCTTTTTCCATGTT	109	120.30	0.9999
		TCAGCTGCTCGTCTAGTTGATG			
*UFD1*	XM_016828251	TGTCAGCCGTTCTAAGGAAACA	107	115.58	0.9773
		ACTTTCTCCCGGTGAATGGC			

^a^ E represents the amplification efficiency of RT-qPCR.

**Table 2:** Expression stability of the 15 candidate reference genes calculated by geNorm (M)^a^.

Rank	Gene	all	Gene	YZ1	Gene	R15	Gene	X33	Gene	X42
1	*18S rRNA*	0.27	*ARF1*	0.24	*ENDO4*	0.25	*ARF2*	0.21	*ARF2*	0.26
2	*ENDO4*	0.27	*18S rRNA*	0.25	*IF4E2*	0.26	*ARF1*	0.21	*18S rRNA*	0.26
3	*ARF1*	0.29	*RPAB5*	0.26	*18S rRNA*	0.27	*ENDO4*	0.21	*UFD1*	0.26
4	*PTBP3*	0.29	*ENDO4*	0.26	*UFD1*	0.29	*18S rRNA*	0.22	*T2FB*	0.26
5	*RPAB5*	0.30	*PTBP3*	0.27	*RPAB5*	0.30	*UFD1*	0.23	*ENDO4*	0.27
6	*IF4E2*	0.30	*IF4E2*	0.28	*ARF2*	0.31	*PTBP3*	0.23	*ARF1*	0.31
7	*UFD1*	0.30	*NUB1*	0.29	*PTBP3*	0.34	*UBE4*	0.27	*PTBP3*	0.32
8	*ARF2*	0.34	*UBE4*	0.31	*UBE4*	0.35	*EF1α*	0.27	*ERF3A*	0.32
9	*ERF3A*	0.34	*EF1α*	0.32	*ARF1*	0.35	*IF4E2*	0.27	*UBC7*	0.33
10	*T2FB*	0.35	*UBC7*	0.33	*ERF3A*	0.35	*RPAB5*	0.28	*IF4E2*	0.34
11	*EF1α*	0.35	*ERF3A*	0.38	*EF1α*	0.36	*UBC7*	0.30	*RPAB5*	0.36
12	*NUB1*	0.38	*UFD1*	0.40	*UBC7*	0.37	*T2FB*	0.32	*NUB1*	0.40
13	*UBC7*	0.38	*ARF2*	0.43	*T2FB*	0.38	*ERF3A*	0.36	*EF1α*	0.41
14	*TAF11*	0.45	*T2FB*	0.45	*NUB1*	0.48	*NUB1*	0.37	*TAF11*	0.47
15	*UBE4*	0.50	*TAF11*	0.46	*TAF11*	0.52	*TAF11*	0.51	*UBE4*	0.90

^a^ The lower M value indicates the more stable gene expression. Ranking of the gene expression stability in all or each of the four cotton cultivars was measured. A higher ranking represents better gene expression stability.

**Table 3:** Expression stability of the 15 candidate reference genes calculated by NormFinder (Sv)^a^.

Rank	Gene	All	Gene	YZ1	Gene	R15	Gene	X33	Gene	X42
1	*18S rRNA*	0.08	*RPAB5*	0.04	*ENDO4*	0.01	*ENDO4*	0.02	*ARF2*	0.04
2	*ENDO4*	0.09	*ARF1*	0.05	*IF4E2*	0.03	*ARF2*	0.05	*T2FB*	0.06
3	*ARF1*	0.10	*18S rRNA*	0.06	*18S rRNA*	0.05	*18S rRNA*	0.05	*UFD1*	0.08
4	*PTBP3*	0.11	*PTBP3*	0.08	*UFD1*	0.13	*PTBP3*	0.06	*18S rRNA*	0.09
5	*IF4E2*	0.13	*IF4E2*	0.11	*RPAB5*	0.13	*ARF1*	0.06	*ENDO4*	0.10
6	*RPAB5*	0.13	*ENDO4*	0.12	*ARF2*	0.14	*UFD1*	0.09	*ERF3A*	0.11
7	*UFD1*	0.13	*UBC7*	0.15	*UBE4*	0.17	*EF1α*	0.11	*ARF1*	0.12
8	*ARF2*	0.17	*UBE4*	0.17	*PTBP3*	0.18	*IF4E2*	0.14	*UBC7*	0.14
9	*ERF3A*	0.17	*NUB1*	0.17	*ARF1*	0.19	*UBE4*	0.15	*PTBP3*	0.16
10	*EF1α*	0.18	*EF1α*	0.19	*ERF3A*	0.19	*UBC7*	0.15	*EF1α*	0.20
11	*T2FB*	0.18	*UFD1*	0.23	*EF1α*	0.20	*RPAB5*	0.16	*IF4E2*	0.20
12	*UBC7*	0.21	*ERF3A*	0.23	*UBC7*	0.20	*T2FB*	0.20	*RPAB5*	0.22
13	*NUB1*	0.21	*ARF2*	0.27	*T2FB*	0.22	*ERF3A*	0.22	*NUB1*	0.23
14	*TAF11*	0.27	*T2FB*	0.28	*NUB1*	0.32	*NUB1*	0.23	*TAF11*	0.25
15	*UBE4*	0.31	*TAF11*	0.28	*TAF11*	0.35	*TAF11*	0.35	*UBE4*	0.60

^a^ Data were presented as the stability value (Sv) calculated by NormFinder. The lower Sv value indicates the better gene expression stability, and a higher ranking represents preferable gene expression stability.

**Table 4:** Expression stability of the 15 candidate reference genes calculated by BestKeeper.

	Total	YZ1	R15	X33	X42
**Gene (R^a^)**	*TAF11* (0.94)	*TAF11* (0.46)	*TAF11* (0.95)	*TAF11* (0.98)	*TAF11* (0.52)
**CV^b^ ± SD^c^**	1.63 ± 0.35	0.53 ± 0.11	2.23 ± 0.48	1.81 ± 0.39	1.72 ± 0.36
**Gene (R)**	*UBC7* (0.94)	*ARF2* (0.44)	*UBC7* (1.00)	*UBC7* (0.99)	*UBE4* (0.98)
**CV ± SD**	2.18 ± 0.45	0.62 ± 0.12	2.52 ± 0.53	2.94 ± 0.61	2.46 ± 0.48
**Gene (R)**	*T2FB* (0.95)	*T2FB* (0.44)	*ARF1* (1.00)	*END04* (1.00)	*UBC7* (0.95)
**CV ± SD**	2.74 ± 0.59	0.75 ± 0.16	2.65 ± 0.50	3.23 ± 0.69	2.49 ± 0.51
**Gene (R)**	*PTBP3* (0.98)	*UBC7* (0.94)	*PTBP3* (1.00)	*EF1α* (0.99)	*EF1α* (0.93)
**CV ± SD**	2.99 ± 0.57	0.94 ± 0.19	2.72 ± 0.52	3.29 ± 0.64	2.83 ± 0.54
**Gene (R)**	*18S rRNA* (0.98)	*RPAB5* (0.98)	*T2FB* (0.96)	*T2FB* (0.97)	*ARF1* (0.99)
**CV ± SD**	3.09 ± 0.64	1.48 ± 0.28	2.91 ± 0.63	3.33 ± 0.73	3.05 ± 0.57
**Gene (R)**	*ENDO4* (0.99)	*UFD1* (0.67)	*18S rRNA* (0.99)	*PTBP3* (1.00)	*T2FB* (1.00)
**CV ± SD**	3.11 ± 0.66	1.63 ± 0.33	2.97 ± 0.62	3.40 ± 0.66	3.05 ± 0.65
**Gene (R)**	*ARF1* (0.98)	*PTBP3* (0.97)	*END04* (1.00)	*18S rRNA* (1.00)	*ARF2* (1.00)
**CV ± SD**	3.17 ± 0.60	1.67 ± 0.32	3.21 ± 0.69	3.70 ± 0.78	3.39 ± 0.64
**Gene (R)**	*UFD1* (0.96)	*18S rRNA* (0.99)	*IF4E2* (1.00)	*ARF2* (1.00)	*PTBP3* (0.98)
**CV ± SD**	3.35 ± 0.70	1.69 ± 0.35	3.25 ± 0.65	3.89 ± 0.76	3.47 ± 0.65
**Gene (R)**	*EF1α* (0.95)	*ARF1* (0.99)	*RPAB5* (0.99)	*UFD1* (1.00)	*UFD1* (0.99)
**CV ± SD**	3.36 ± 0.64	1.82 ± 0.34	3.66 ± 0.70	3.92 ± 0.83	3.53 ± 0.73
**Gene (R)**	*RPAB5* (0.98)	*END04* (1.00)	*UFD1* (0.67)	*NUB1* (0.98)	*ENDO4* (1.00)
**CV ± SD**	3.49 ± 0.66	1.95 ± 0.41	3.70 ± 0.78	3.98 ± 0.84	3.54 ± 0.75
**Gene (R)**	*UBE4* (0.92)	*IF4E2* (1.00)	*UBE4* (0.99)	*UBE4* (1.00)	*18S rRNA* (0.99)
**CV ± SD**	3.54 ± 0.71	2.08 ± 0.41	3.91 ± 0.80	4.05 ± 0.83	3.63 ± 0.74
**Gene (R)**	*IF4E2* (0.99)	*NUB1* (0.95)	*ARF2* (1.00)	*ARF1* (1.00)	*ERF3A* (0.98)
**CV ± SD**	3.55 ± 0.70	2.18 ± 0.44	3.96 ± 0.77	4.12 ± 0.79	3.73 ± 0.69
**Gene (R)**	*ARF2* (0.96)	*UBE4* (0.99)	*EF1α* (0.99)	*RPAB5* (0.98)	*IF4E2* (0.99)
**CV ± SD**	3.66 ± 0.70	2.24 ± 0.45	4.66 ± 0.89	4.22 ± 0.81	3.97 ± 0.77
**Gene (R)**	*NUB1* (0.98)	*EF1α* (0.94)	*NUB1* (1.00)	*IF4E2* (0.99)	*RPAB5* (0.99)
**CV ± SD**	3.85 ± 0.80	2.44 ± 0.46	4.69 ± 0.98	4.24 ± 0.85	4.09 ± 0.77
**Gene (R)**	*ERF3A* (0.98)	*ERF3A* (0.99)	*ERF3A* (0.99)	*ERF3A* (0.98)	*NUB1* (0.98)
**CV ± SD**	3.98 ± 0.74	2.72 ± 0.50	4.74 ± 0.89	4.35 ± 0.82	4.18 ± 0.86

^a^ R: correlation coefficient of the Cq (quantification cycle) level; ^b^ CV: coefficient of variance expression as a percentage of the Cq level; ^c^ SD: standard deviation of the Cq. The lower values of CV and SD indicate the better expression stability of the reference genes.

## Supplementary Materials

The following are available online at https://www.mdpi.com/2073-4425/10/7/497/s1, Figure S1: Melting curves of the 15 selected reference genes, Table S1: Cq value of the 15 candidate reference genes, Table S2: The details of the 15 candidate reference genes based on RNA-Seq data.
