# Supplementary material for: Screening the Reference Genes for Quantitative Gene Expression by RT-qPCR During SE Initial Dedifferentiation in Four Gossypium hirsutum Cultivars that Have Different SE Capability
Source: Genes (Basel). 2019 Jun 28;10(7):497. doi: 10.3390/genes10070497 (PMC6678594; doi:10.3390/genes10070497)
Supplement: Supplementary file 1 [file genes-10-00497-s001.zip › Supplementary files-R1/Table S1.docx]

Table S1. Cq value of the 15 candidate reference genes

|  | YZ1-  0h | YZ1-3h | YZ1-3d | R15-0h | R15-3h | R15-3d | X33-0h | X33-3h | X33-3d | X42-0h | X42-3h | X42-3d |
| --- | --- | --- | --- | --- | --- | --- | --- | --- | --- | --- | --- | --- |
| 18S rRNA | 20.98 | 20.42 | 19.99 | 21.72 | 20.72 | 19.95 | 22.05 | 21.24 | 19.89 | 20.75 | 21.02 | 19.22 |
| ARF1 | 19.01 | 18.48 | 18.02 | 19.76 | 18.90 | 18.35 | 20.01 | 19.47 | 17.96 | 18.72 | 19.24 | 17.71 |
| ARF2 | 18.70 | 18.90 | 18.58 | 20.67 | 19.50 | 18.37 | 20.41 | 19.89 | 18.44 | 19.39 | 19.60 | 18.05 |
| EF1α | 19.68 | 18.80 | 18.47 | 20.50 | 18.85 | 18.13 | 20.23 | 19.34 | 18.35 | 19.51 | 19.22 | 18.16 |
| ENDO4 | 21.56 | 21.10 | 20.41 | 22.61 | 21.52 | 20.59 | 22.38 | 21.65 | 20.45 | 21.59 | 21.82 | 20.02 |
| ERF3A | 19.15 | 18.34 | 17.70 | 20.04 | 18.47 | 17.63 | 20.18 | 18.93 | 17.72 | 18.95 | 18.85 | 17.36 |
| IF4E2 | 20.05 | 19.65 | 18.94 | 20.93 | 19.94 | 19.00 | 21.04 | 20.59 | 18.89 | 19.69 | 20.32 | 18.27 |
| NUB1 | 21.05 | 20.21 | 19.89 | 22.45 | 20.76 | 19.72 | 22.29 | 21.02 | 19.79 | 21.14 | 21.10 | 19.19 |
| PTBP3 | 19.19 | 19.04 | 18.40 | 20.01 | 19.32 | 18.49 | 20.37 | 19.60 | 18.49 | 19.02 | 19.68 | 17.88 |
| RPAB5 | 19.05 | 18.84 | 18.32 | 20.11 | 19.25 | 18.10 | 19.98 | 19.69 | 18.01 | 19.01 | 19.71 | 17.63 |
| T2FB | 21.29 | 21.57 | 21.13 | 22.46 | 21.97 | 20.79 | 22.46 | 22.33 | 20.76 | 21.73 | 22.16 | 20.47 |
| TAF11 | 21.47 | 21.15 | 21.33 | 22.39 | 21.29 | 21.31 | 22.38 | 21.74 | 21.24 | 21.38 | 21.57 | 20.66 |
| UBE4 | 20.64 | 19.83 | 19.42 | 21.66 | 20.07 | 19.64 | 21.73 | 20.73 | 19.35 | 20.39 | 19.32 | 19.28 |
| UBC7 | 20.82 | 20.43 | 20.35 | 21.74 | 20.74 | 20.35 | 21.23 | 20.82 | 19.66 | 20.53 | 20.98 | 19.61 |
| UFD1 | 20.57 | 20.82 | 19.94 | 22.23 | 21.01 | 19.94 | 22.01 | 21.42 | 19.85 | 21.02 | 21.32 | 19.54 |
