# Supplementary figures and images for: Screening the Reference Genes for Quantitative Gene Expression by RT-qPCR During SE Initial Dedifferentiation in Four Gossypium hirsutum Cultivars that Have Different SE Capability
Source: Genes (Basel). 2019 Jun 28;10(7):497. doi: 10.3390/genes10070497 (PMC6678594; doi:10.3390/genes10070497)

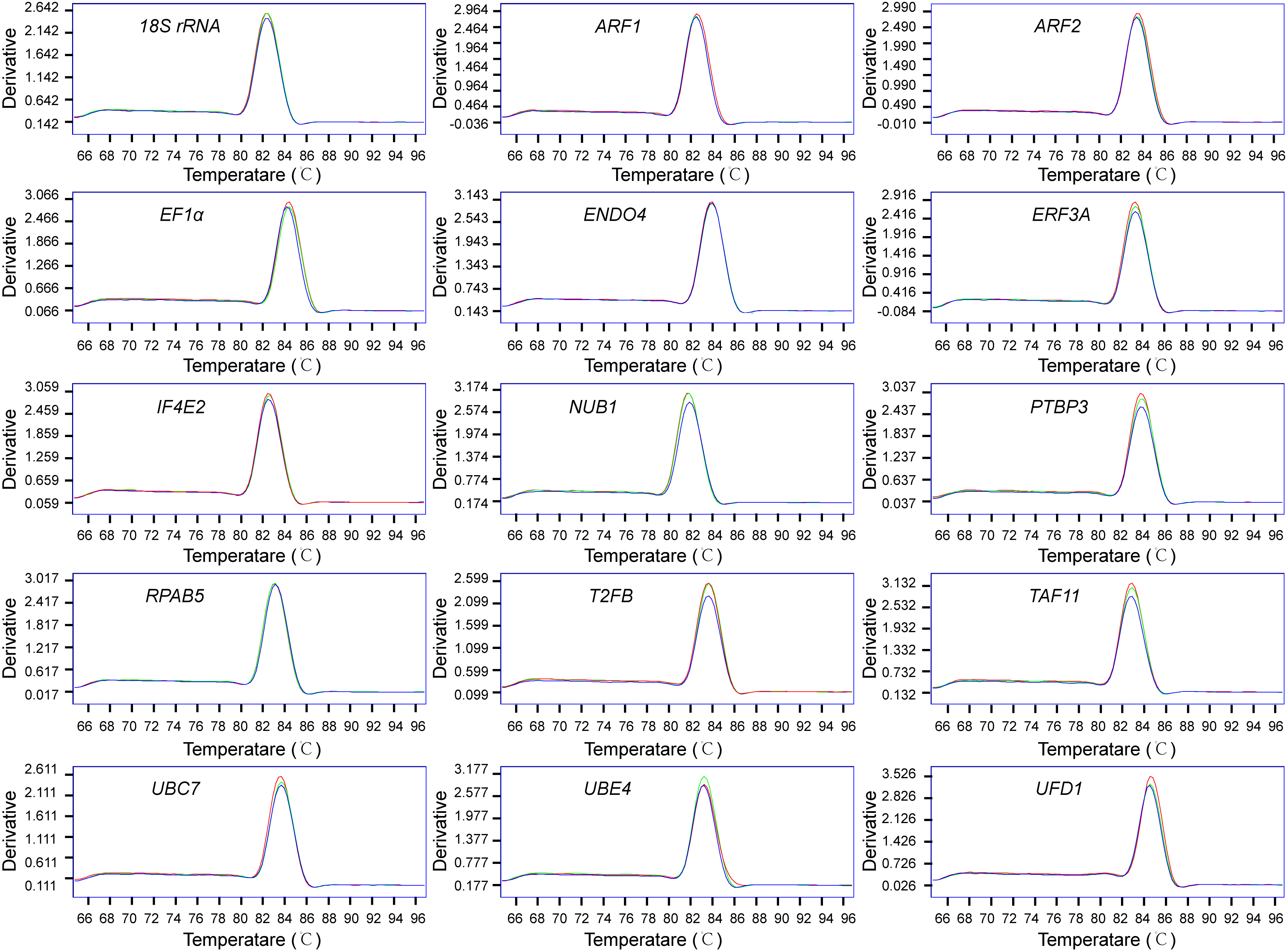

Supplement: Supplementary file 1 [file genes-10-00497-s001.zip › Supplementary files-R1/Figure S1.tif]
